# A Survey of Chemical Compositions and Biological Activities of Yemeni Aromatic Medicinal Plants

**DOI:** 10.3390/medicines2020067

**Published:** 2015-05-28

**Authors:** Bhuwan K. Chhetri, Nasser A. Awadh Ali, William N. Setzer

**Affiliations:** 1 Department of Chemistry, University of Alabama in Huntsville, Huntsville, AL 35899, USA; E-Mail: bkc0007@uah.edu; 2 Pharmacognosy Department, Faculty of Pharmacy, Sana’a University, P.O. Box 13150 Sana’a, Yemen; 3 Pharmacognosy Department, Faculty of Clinical Pharmacy, Albaha University, P.O. Box 1988 Al Baha, Saudi Arabia

**Keywords:** aromatic plants, herbal medicine, essential oil compositions, Asteraceae, Burseraceae, Lamiaceae, Yemen

## Abstract

Yemen is a small country located in the southwestern part of the Arabian Peninsula. Yemen’s coastal lowlands, eastern plateau, and deserts give it a diverse topography, which along with climatic factors make it opulent in flora. Despite the introduction of Western medicinal system during the middle of the twentieth century, herbal medicine still plays an important role in Yemen. In this review, we present a survey of several aromatic plants used in traditional medicine in Yemen, their traditional uses, their volatile chemical compositions, and their biological activities.

## 1. Introduction

From prehistoric times people have been using medicinal plants for the treatment of a wide variety of ailments. This traditional use of plants is based upon an empirical, timeless trial and error, correlating certain plants to the management and cure of particular diseases. These plants were used as herbal formulations in crude forms like tinctures, teas, powders, and poultices. The traditional way by which these plants were used can still be found in communities, passed down through natural history, and still prevail to be a successful form of medication, despite the advent of modern medicine.

Aromatic medicinal plants are plants with aroma characteristics as well as having medicinal properties and are “chemical goldmines” because of the diverse range of secondary metabolites that they possess and the wide range of pharmacological activities that they show [1]. The use of aromatic medicinal plants has been increasing steadily with notable use in the pharmaceutical, cosmetic, and food industries [2].

**Figure 1:**
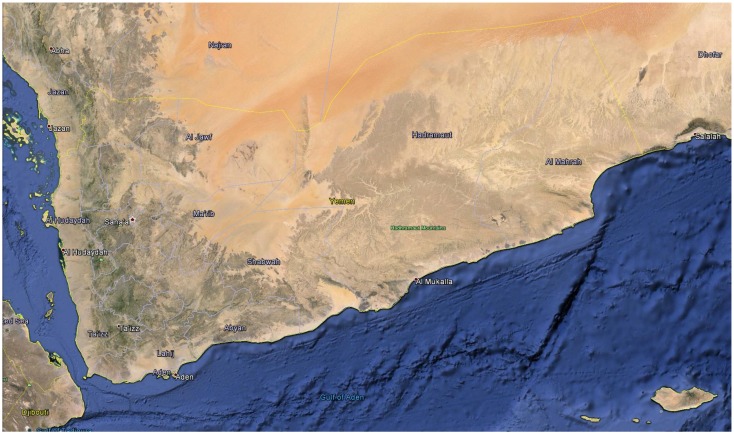
Google Earth^©^ satellite view of Yemen.

The republic of Yemen was formed in 1990 by merging the northern and southern states of Yemen [3]. It is located in the southwest part of Arabian Peninsula, between 12°40′ and 19°00′ N latitude and 42°30′ to 53°05′ E longitude (see Figure 1) [4]. Unlike the Arabian Peninsula, which is known for its vast inhospitable deserts, Yemen has rugged highlands and mountains with several peaks that are more than 3000 m (10,000 ft). The Yemeni highlands experience relatively abundant rainfall and have a temperate climate. Yemen’s coastal lowlands, eastern plateau and deserts give it a high topographic diversity, which along with climatic factors, has resulted in a diverse and rich flora. Yemen occupies a very vital position on the southern Arabian Peninsula sharing borders with two countries, Saudi Arabia to the north and Oman towards the northeast. The east and south of Yemen border the Red Sea and the Gulf of Aden with a total coastline measuring 1906 km (1184 miles). Yemen also has more than 100 small islands scattered in the nearby Red Sea and Arabian Sea. The total area of Yemen including these islands is 527,970 sq km (203,850 sq mi) [4].

There are two major forces that determine the climate of Yemen. Winters in Yemen are dominated by dry northerly winds whereas moist monsoons prevail in spring and summer. The other major factor that is responsible for the climatic condition is the elevation, with the highlands experiencing a temperate climate with mild, dry winters and warm summers with abundant rainfall. To take an example, Sana’a, a region in the central highlands, has an average temperature of 14 °C in January and 22 °C in July. The average annual rainfall in Sana’a is 265 mm (10.4 in), whereas the highlands around Ibb and Taiz receive more than 1000 mm (40 in) each year. Sana’a is the capital and the largest city of Yemen. At an altitude of more than 2200 m, the city extends across a very fertile basin near the foot of the mountain called Jabal Nuqum. Taiz is at an altitude of 1400 m in the fertile highlands. It is a productive agricultural region where coffee (*Coffea arabica*) and khat (*Catha edulis*), as mild stimulants, are the main crops. Ibb is also in the southern highlands, north of Taiz at an elevation of 2050 m. The area gets abundant rainfall and has rich volcanic soil, which makes it green and agriculturally productive, the chief crops being grains, coffee, khat, fruits and vegetables [4].

**Figure 2:**
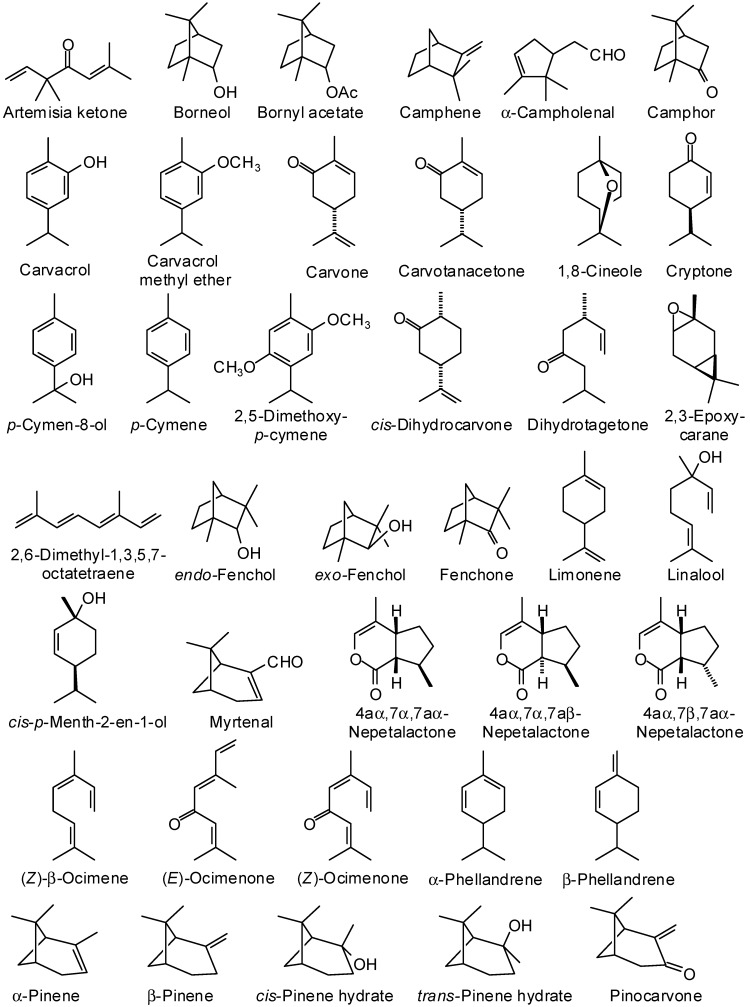

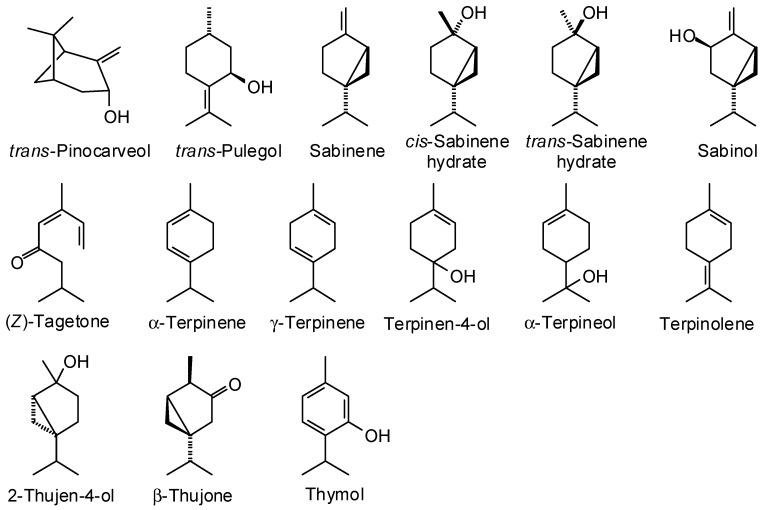
Monoterpenoid essential oil components discussed in this review.

**Figure 3:**
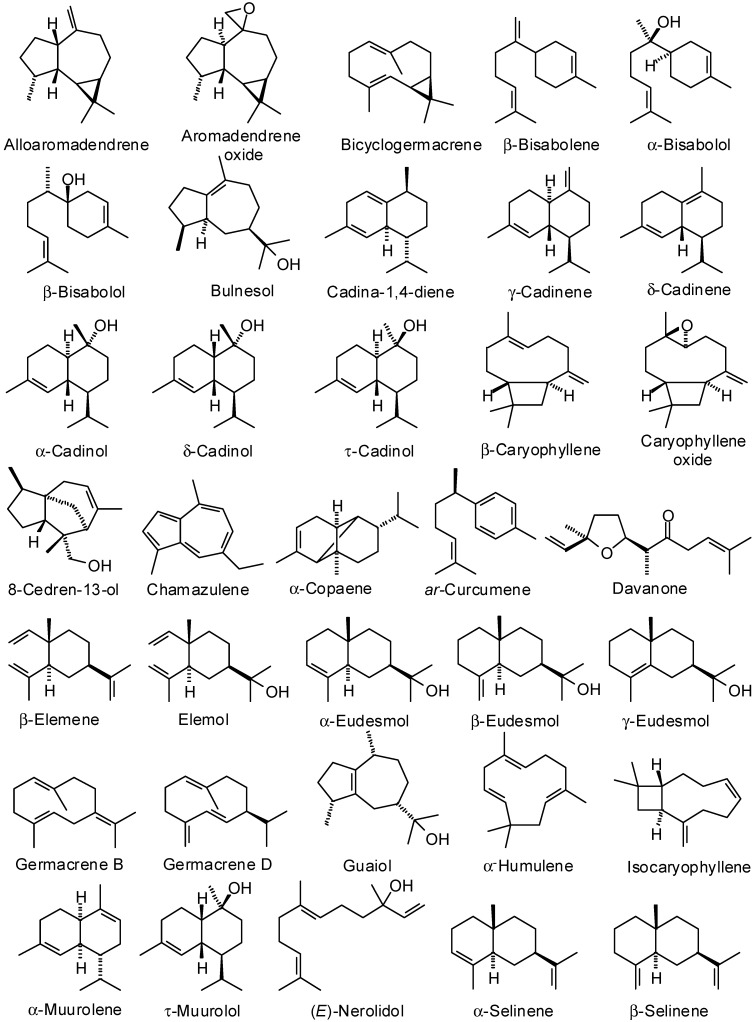

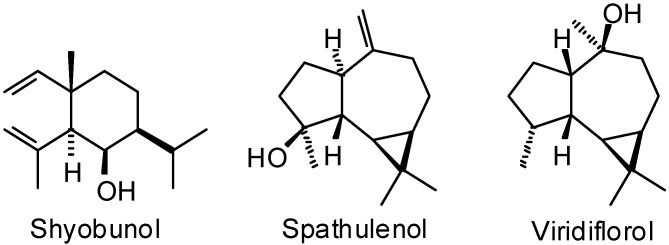
Sesquiterpenoid essential oil components discussed in this review.

**Figure 4:**
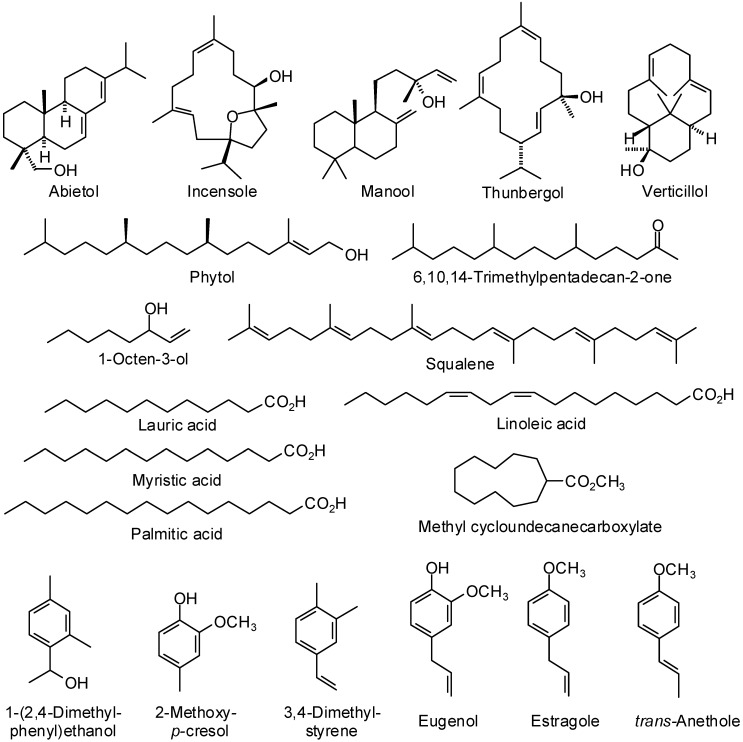
Miscellaneous essential oil components discussed in this review.

There has been an ever-increasing interest in natural products as alternatives for artificial additives of pharmacologically relevant agents in recent years [5], and essential oils from aromatic plants have gained much interest for their use in various foods, cosmetics, and pharmaceutical products. They have widespread use as flavoring materials and stand as “green” alternatives in pharmaceutical, agricultural, nutritional, and many other fields. They show a wide range of biological activities, which account for the use of aromatic plants in traditional medicine and for their growing interest as alternative therapies for the prevention, cure, and alleviation of certain diseased conditions [1,6]. Essential oils have been widely studied and used for their antimicrobial, antiviral, nematicidal, antifungal, insecticidal and antioxidant properties. In this review, we have focused on the traditional medicinal uses and volatile components (Figure 2, Figure 3 and Figure 4) of several aromatic medicinal plants from Yemen.

## 2. Aromatic Medicinal Plants from Yemen

### 2.1. Anacardiaceae

#### *Schinus molle* L.

There are around 33 species of *Schinus*, all of which are native to the Neotropics [7]. There is only one species, *Schinus molle,* which has been introduced to Yemen. The plant, known as “filfil katheb”, is used in Yemeni folk medicine as an expectorant, a diuretic and also for the treatment of stomach upsets [8]. Bioactivity studies of *S. molle* essential oil by various researchers have shown that it possesses antibacterial, antifungal [9], cytotoxic [10], insecticidal, and insect repellent [11] properties. The essential oil of *S. molle* from Yemen, obtained by hydrodistillation contained β-caryophyllene (13.5%), α-pinene (2.8%), carvacrol (2%), germacrene D (16.7%), δ-cadinene (3.2%), spathulenol (3.4%), caryophyllene oxide (6.7%), viridiflorol (3.3%), α-cadinol (2.5%), α-bisabolol (4%), and an unknown compound (18%). The same plant was also extracted by supercritical CO_2_ and analyzed by GC-MS. Here the major components were comparable with only with some minor differences in their concentrations. The notable compounds identified were α-pinene (1.6%), β-caryophyllene (9.1%), germacrene D (13.7%), bicyclogermacrene (2%), spathulenol (2.7%), shybunol (3.3%), abietol (5.0%) and an unknown compound (37%) [12]. The observed bioactivities of *S. molle* essential oil can be attributed to the relatively large concentrations of the sesquiterpenoids β-caryophyllene, germacrene D, and caryophyllene oxide, which have shown antimicrobial and cytotoxic activities [13].

### 2.2. Asteraceae

#### 2.2.1. *Artemisia abyssinica* Sch. Bip. ex A. Rich.

There are around 400 species of *Artemisia*, distributed typically in Asia, Europe, and North America [7]. *Artemisia abyssinica*, known as “boitheran” in Yemen, is an aromatic, grey, silky-hairy plant with pale yellow flower-heads and is well known as a stimulant and an analgesic. It is short lived perennial plant, with sparingly branched stems that are grooved especially above. Leaves are alternate, grey-green, deeply bipinnatisect with linear segments, and 4–10 cm long. It is widely spread on the high plateau from 2200 to 3600 m and often abundant on roadsides, alluvial plains, and abandoned fields. It is used in Yemen for treating headache and as insect repellent. In Saudi Arabia, a decoction of fresh whole plant is traditionally used to treat diabetes mellitus [14]. The plant has also been used in folk medicine as an anthelmintic, antispasmodic, antirheumatic and antibacterial agent [15]. Antioxidant, antileishmanial and antitrypanosomal activities have also been recorded for *Artemisia abyssinica* essential oil [15]. Essential oil compositions have been determined for *Artemisia abyssinica* from three different regions of Yemen, namely Taiz (higher than 1500 m), Sana’a (higher than 3000 m) and Alhodiadah (coastal region). The major components of *Artemisia abyssinica* essential oils from Yemen are listed in Table 1 [16,17]. *Artemisia abyssinica* essential oil was rich in camphor and davanone with lesser amounts of (*E*)-nerolidol, *cis*-sabinene hydrate, terpinen-4-ol, linalool, and bornyl acetate. *Artemisia abyssinica* essential oil showed marginal cytotoxic activity against MCF-7 cells (30% cell viability reduction at a concentration of 100 μg/mL) [16]. *Artemisia abyssinica* oil from Yemen is remarkably different in composition from that reported from Ethiopia, which was rich in yomogi alcohol (38.5%), artemisyl acetate (24.9%), and artemisia alcohol (6.7%) [18]. 

**Table 1:** Major components (%) in essential oils of *Artemisia abyssinica* from Yemen.

Compound	Taiz (above 1500 m)	Sana’a (above 3000 m)	Alhodiadah (Coastal)
α-Pinene	1.1	0.5	1.1
*cis-*Sabinene hydrate	2.8	3.6	5.8
Linalool	2.1	2.7	1.6
Camphor	29.6	42.1	42.5
Terpinen-4-ol	2.0	3.8	3.9
Bornyl acetate	1.3	2.3	2.3
(*E*)-Nerolidol	5.1	4.0	4.5
Davanone	49.4	34.5	32.3

#### 2.2.2. *Artemisia arborescens* L.

*Artemisia arborescens* has been used traditionally as an anti-inflammatory remedy. The essential oil of *Artemisia arborescens* has been reported to have antibacterial [19] and antifungal activities [15] as well as antiviral activity against HSV-1 and HSV-2 [20]. The essential oil of *Artemisia arborescens* from Yemen has been reported to have α-terpinene (8.7%), artemisia ketone (51.5%), camphor (14.1%), α-bisabolol (12.6%) and palmitic acid (2.4%) as the major constituents [21]. The oil showed strong *in vitro* cytotoxic activity with an *IC*_50_ of 16.9 µg/mL against HT 29 human colorectal adenocarcinoma cells. *Artemisia arborescens* essential oil also exhibited antifungal activity against *Cladosporium cucumerinum*. The cytotoxic activity of *Artemisia arborescens* can be attributed to the presence of α-bisabolol [22] and palmitic acid [23]. The essential oil composition of *Artemisia arborescens* from Yemen [21] was very different from samples from Sicily [19] or Algeria [24], which were dominated by β-thujone and chamazulene. 

#### 2.2.3. *Conyza bonariensis* (L.) Cronquist

*Conyza bonariensis* (L.) Cronquist is known as “sadaf” in Yemen. It is an annual or short lived perennial weed. A variety of phytochemicals have been reported from the genus that includes alkaloids, volatile oils, terpenoids, phenolic acids, flavonoids and hydrolyzable tannins. *Conyza bonariensis* is widely used in folk medicine for the treatment of rheumatism, cystitis, gout, nephritis, dysmenorrhea, tooth pain and headache. Other studied biological activities of *Conyza bonariensis* include molluscicidal activity against *Biomphalaria* snails, anti-inflammatory activity, antipyretic effects, and antimicrobial activity [25]. Essential oil from the aerial plant parts of *Conyza bonariensis* collected from Yemen was dominated by the sesquiterpenoids 8-cedren-13-ol (18.5%) and aromadendrene oxide (18.8%) [26].

#### 2.2.4. *Pulicaria inuloides* (Poir.) DC.

There are 77 species of *Pulicaria*, distributed in temperate and warm Eurasia [7], and in folk medicine *Pulicaria* species have been used as insect repellents, galactagogues, antiepileptics, and as tonics [27]. *Pulicaria inuloides* is distributed from Morocco east throughout North Africa, south to Chad, Niger, and Somalia, and east to the Arabian Peninsula [28]. The plant, known as “sekba”, is used traditionally in Yemen to treat wounds [29]. The essential oil of *Pulicaria inuloides* from Yemen was composed largely of carvotanacetone (47.3%) and palmitic acid (12.8%) [30]. The oil has demonstrated antimicrobial activity against *Staphylococcus aureus*, *Streptococcus pneumoniae*, *Bacillus subtilis*, *Escherichia coli*, and *Candida albicans*, and antioxidant activity in the 2,2-diphenyl-1-picrylhydrazyl (DPPH) radical-scavenging and β-carotene bleaching assays [31].

#### 2.2.5. *Pulicaria jaubertii* Gamal-Eldin

*Pulicaria jaubertii* is a perennial fragrant herb with erect branches growing up to 50 cm [32]. The plant, known as “anssif” in Yemen, is distributed in the southern Arabian Peninsula, and is used traditionally as a diuretic and antipyretic. Leaves of *Pulicaria jaubertii* are also used flavoring agents for cooking. Similar to *Pulicaria inuloides* (above), the essential oil of *Pulicaria jaubertii* from Yemen was dominated by carvotanacetone (64.0%) [33]. The essential oil of *Pulicaria jaubertii* from southern Saudi Arabia had a higher proportion of carvotanacetone (98.6%), and this essential oil had excellent *in vitro* cytotoxic activity against MCF-7 and Hep-G2 cells (*IC*_50_ = 3.8 and 5.1 μg/mL, respectively), but was only marginally antibacterial [32].

#### 2.2.6. *Pulicaria stephanocarpa* Balf. f.

On Soqotra island of Yemen, *Pulicaria* is represented by seven species, some of which have traditional importance as antiseptic agents and as food additives [34]. *Pulicaria stephanocarpa*, known as “derbeb” in Soqotra, has been traditionally used in a variety of health conditions including headache, abscesses, boils and sores. The essential oil of *Pulicaria stephanocarpa* from Soqotra has a high percentage of oxygenated sesquiterpenoids. One study showed the oil to be rich in α-cadinol (42.5%), spathulenol (22.0%), and β-caryophyllene (10.8%) [35], while a second revealed the major constituents to β-caryophyllene (13.4%), (*E*)-nerolidol (8.5%), caryophyllene oxide (8.5%), α-cadinol (8.2%), spathulenol (6.8%), and τ-cadinol (4.7%) [34]. The essential oil had moderate to high antimicrobial activity against *Staphylococcus aureus* (MIC= 3.12 µL/mL), *Bacillus subtilis* (12.5 µL/L) and *Candida albicans* (6.25 µL/mL). The oil showed free radical scavenging activity with an *IC*_50_ of 330 µg/mL as assessed by DPPH assay. Furthermore, the oil was tested for acetylcholine esterase (AChE) inhibitory activity where it showed an inhibition of 47% at concentration of 200 µg/mL using Ellman’s method [34].

#### 2.2.7. *Pulicaria undulata* (L.) C.A. Mey.

An aromatic tea of *Pulicaria undulata*, known as “kho’ah”, is used in the central Sahara to treat chills, diabetes, cardiac disorders, skin diseases, and abscesses [36], and in Egypt to treat inflammation, as an insect repellent, and an herbal tea [37]. *Pulicaria undulata* oil has shown antibacterial [38], sedative [39], and insecticidal activities [40]. *Pulicaria undulata* essential oil from Yemen has been reported to have an extremely high concentration of carvotanacetone (91.4%) along with 2,5-dimethoxy-*p*-cymene (2.6%) [41]. Although carvotanacetone has been found in *Pulicaria undulata* oil collected from different geographical regions, comparison shows that the highest concentration is found in Yemeni *Pulicaria undulata*. Thus, for example, *Pulicaria undulata* oil from Sudan was composed of 55.9% carvotanacetone [42].

The essential oil from Yemen did show moderate *in vitro* cytotoxic activity on MCF-7 breast tumor cells (*IC*_50_ = 64.6 μg/mL), which can be attributed to the high concentration of carvotanacetone (see above). The oil also exhibited antibacterial activity against *Staphylococcus aureus* with minimum bactericidal concentration (MBC) of 3.12 µL/mL for *Staphylococcus aureus* and methicillin-resistant *Staphylococcus aureus*. The MBC against *Candida albicans* and *Bacillus subtilis* was 6.25 µL/mL, whereas MBC against *Escherichia coli* was 12.5 µL/mL [41].

#### 2.2.8. *Tagetes minuta* L.

*Tagetes minuta* is native to southern South America, but it has been introduced to Europe, Asia, the Middle East, and Africa [43]. The plant is known as “nirgis” in Yemen and used traditionally as a tea to treat the common cold, upper and lower respiratory inflammations, and for digestive ailments [43]. *Tagetes minuta* essential oil is an article of commerce and is used in baked goods, candy, ice cream, and soft drinks [44]. *Tagetes minuta* is used in the Arabian Peninsula as an antimicrobial, anthelmintic, diuretic, and antispasmodic [45], and the essential oil has shown biocidal, acaricidal, antifungal and antimicrobial activities [46,47,48,49,50,51]. The essential oil of *Tagetes minuta* from Yemen has been reported to have (*Z*)-ocimenone (15.9%), (*E*)-ocimenone (34.8%), (*Z*)-β-ocimene (8.3%), limonene (2.3%), (*Z*)-tagetone (1.8%), and dihydrotagetone (1.4%). The oil had moderate cytotoxic activity against MCF-7 cancer cell line with an *IC*_50_ of 54.7 µg/mL. However, the oil had a potent DPPH radical-scavenging activity with an *IC*_50_ of 36 µg/mL. Moreover, the oil was highly active against methicillin-resistant *Staphylococcus aureus* (MRSA) [51]. Similarly, *Tagetes minuta* essential oil showed considerable antioxidant activity in the thiobarbituric acid reactive substances (TBARS) assay [52].

#### 2.2.9. *Tarchonanthus camphoratus* L.

*Tarchonanthus camphoratus*, known locally as “bayad” or “mukar” in Yemen, is a strongly aromatic shrub found growing in the hillsides between 1200–2500 m [53]. Traditionally, its aromatic leaves are used for wounds and urinary tract infections [29]. An infusion of the leaves is used to treat stomach ailments and bronchitis. Burning of the aerial parts of the plant liberates smoke that is inhaled for the treatment of sinus-related complaints and for headaches. The burned seeds and leaves are used traditionally as fumigating agents in funeral rituals. Leaves can also be used as hot poultices for treating chest complaints whereas chewing the leaves is believed to alleviate toothache. There have been numerous studies on the essential oil of *Tarchonanthus camphoratus* and a wide variety of biological properties (e.g., antimicrobial, antifungal, antioxidant, antidiabetic, insecticidal, insect repellant, analgesic, and antipyretic) have been reported [53]. *Tarchonanthus camphoratus* leaf essential oil from Yemen is composed largely of oxygenated monoterpenoids (48.3%) and oxygenated sesquiterpenoids (32.7%). The principle compounds were *endo*-fenchol (21.2%), *trans*-pinene hydrate (8.8%), caryophyllene oxide (7.5%), α-terpineol (6.4%), τ-cadinol (6.4%) and α-cadinol (5.2%). The essential oil was also reported to have moderate antimicrobial activity in disc diffusion assay against a number of pathogenic bacteria including methicillin-resistant *Staphylococcus aureus* (MRSA) and *Candida albicans*. The oil also showed moderate *in vitro* cytotoxic activity against HT29 (human colonic adenocarcinoma) tumor cells with an *IC*_50_ of 84.7 µg/mL [53].

### 2.3. Burseraceae

#### 2.3.1. *Boswellia ameero* Balf. f.

The genus *Boswellia* belongs to the Burseraceae and has about 24 species [7]. They are typically small trees and are found to grow mainly in Arabia, on eastern coast of Africa and in India. *Boswellia* trees contain a natural oleogum resin (frankincense) that exudes upon scoring the bark of the tree. On Soqotra Island of Yemen, there are eight endemic *Boswellia* species. Oleogum resin has been used traditionally by the native inhabitants for relieving toothache pain, sweetening the breath, and to sooth a disturbed stomach [54]. *Boswellia ameero* is endemic to Soqotra and is known locally as “ameero”. It is dominant in woodlands on granite substrata above 600 m [55]. *Boswellia ameero* essential oil is composed of (3*E*,5*E*)-2,6-dimethyl-1,3,5,7-octatetraene (34.9%), 1-(2,4-dimethylphenyl)-ethanol (20.3%), 3,4-dimethylstyrene (17.3%), α-campholenal (13.4%), and α-terpineol (12.4%) and as the major components [56]. This oil showed DPPH radical-scavenging activity (*IC*_50_ = 175.2 µg/mL) and AChE enzyme inhibitory activity (41.5% inhibition at 200 μg/mL).

#### 2.3.2. *Boswellia elongata* Balf. f.

On Soqotra, the endemic *Boswellia elongata* is found on the limestone plateau between 300 and 450 m, on stony soils, and is the *Boswellia* species producing the most valuable frankincense [55]. Both *Boswellia* *ameero* and *Boswellia elongata* are used on Soqotra to treat the common cold, bronchitis, asthma, and rheumatism [57]. The essential oil of *Boswellia elongata* was found to be dominated by verticillol (52.4%), β-caryophyllene (39.1%), and methyl cycloundecanecarboxylate (7.9%) [56]. *Boswellia elongata* essential oil showed relatively weak DPPH radical-scavenging activity (*IC*_50_ = 211 μg/mL) and AChE enzyme inhibitory activity (29.6% inhibition at 200 μg/mL).

#### 2.3.3. *Boswellia socotrana* Engl.

Another *Boswellia* endemic to Soqotra, *Boswellia socotrana*, known as “samahno”, is found primarily on the coastal plain of the island [55]. The essential oil was composed of (*E*)-2,3-epoxycarane (51.8%), 2-thujen-4-ol (31.3%), *p*-cymene (7.1%), 4-terpinenyl acetate (3.9%), and *p*-menth-1(7)-en-2-one (2.6%) [56]. Of the *Boswellia* essential oils tested, *Boswellia socotrana* showed the best DPPH radical-scavenging (*IC*_50_ = 211 μg/mL) and AChE inhibitory (59.3% inhibition at 200 μg/mL) activities.

#### 2.3.4. *Commiphora habessinica* (O. Berg) Engl.

*Commiphora* is also a member of the Burseraceae and the genus has more than 150 species [7]. The resinous exudates (myrrh) of *Commiphora* species have long been used in the form of perfumes and incense as well as for health problems such as stomachache, colds, wounds, malaria, fever, and as an antiseptic and against skin infections. *Commiphora habessinica* is known in Yemen as “khdash”. The major components of the hydrodistilled oil of *Commiphora habessinica* oleogum resin from Yemen were β-elemene (32.1%), α-selinene (18.9%), cadina-1,4-diene (7.5%), germacrene B (3.6%), α-copaene (3.5%), τ-muurolol (3.0%), caryophyllene oxide (2.9%), α-cadinol (2.6%) [58].

#### 2.3.5. *Commiphora kua* Vollesen

The Soqotra island of Yemen is represented by five species of *Commiphora*, namely *Commiphora socotrana*, *Commiphora planifrons*, *Commiphora parvifolia*, *Commiphora ornifolia*, and *Commiphora kua* [59]. In Yemeni traditional medicine, the powdered resin of *Commiphora kua* (“bisham”) is administered with warm milk or water for stomachache in young children [60]. The leaves of this plant are used to cleanse the mouth and throat, to treat cough and bronchitis, and as an antiseptic on the skin [61]. The major constituents of the hydrodistilled *Commiphora kua* resin from Soqotra were α-cadinol (33.0%), γ-cadinene (22.5%), δ-cadinene (17.0%), isocaryophyllene (3.7%), alloaromadendrene (2.8%), α-muurolene (2.7%), and α-humulene (2.4%) [60]. This essential oil showed moderate antifungal activity against *Cladosporium cucumerinum*. 

#### 2.3.6. *Commiphora ornifolia* J.B. Gillett

*Commiphora ornifolia* is endemic to the island of Soqotra and is known as “ikshah”. The bark of *Commiphora ornifolia* is used in Yemeni traditional medicine as an antiseptic, as an emmenagogue, as well as to treat diarrhea and dysentery [62]. The major components of the bark essential oil of *Commiphora ornifolia* were determined to be camphor (27.3%), *endo*-fenchol (15.5%), caryophyllene oxide (6.5%), thunbergol (6.4%), fenchone (4.4%), incensole (3.8%), borneol (2.9%) and manool (2.7%) [63]. *Commiphora ornifolia* bark oil had antibacterial against Gram-positive *Staphylococcus aureus* and *Bacillus subtilis* (MIC = 810 and 400 μg/mL, respectively), but demonstrated only weak DPPH radical-scavenging activity.

#### 2.3.7. *Commiphora parvifolia* Engl.

*Commiphora parvifolia*, known as “likham”, is also endemic to Soqotra and is traditionally used as an antiseptic, uterine stimulant, emmenagogue, and to treat diarrhea and dysentery [57]. The essential oil from bark of *Commiphora parvifolia* showed weak DPPH radical-scavenging activity and antimicrobial activity, and the major components were palmitic acid (18.4%), caryophyllene oxide (14.2%), camphor (9.1%), β-eudesmol (7.7%), phytol (5.8%), bulnesol (5.7%), *endo*-fenchol (3.9%), and τ-cadinol (3.7%) [63].

### 2.4. Celastraceae

#### *Catha edulis* Forssk.

*Catha edulis*, a member of the Celastraceae, is a dicotyledonous evergreen shrub known locally known as “khat”. The major cultivation areas of khat is Ethiopia, particularly the Harar district, and Yemen. Chewing khat is a primary recreation in Yemen [4,64]. *Catha edulis* leaves contain several chemical groups of compounds including alkaloids, tannins, flavonoids, terpenes and sterols, and essential oils. Many studies have confirmed the presence of a complex set of alkaloids called kathdulinat, which are responsible for its stimulant effects especially phenylalkylamine compounds (cathinone and cathine) [65,66]. The leaves of khat are chewed to increase activity and elevate the mood. The roots and leaves are used traditionally for the treatment of influenza, cough, gonorrhea, chest and stomach problems [67]. The essential oil of *C. edulis* leaves from Yemen has been reported to have a high concentration of carvotanacetone (84.4%) in addition to *trans*-pulegol (2.2%) and 2,5-dimethoxy-*p*-cymene (1.9%) [67].

### 2.5. Lamiaceae

#### 2.5.1. *Ajuga bracteosa* Wall. ex Benth.

The Lamiaceae is a family of great importance due to its extensive use in folk medicine, cosmetics, culinary, and for the commercial production of essential oils [68]. *Ajuga bracteosa* is known locally as “hodam” and is a traditionally used medicinal plant that belongs to the Lamiaceae. The genus *Ajuga* is represented by only one species, *Ajuga bracteosa*, in Yemen. It is an erect and aromatic perennial herb used traditionally in Yemeni medicine as an antiseptic agent and to treat toothache [29]. The essential oil of *Ajuga bracteosa* from Yemen has been reported with high concentrations of oxygenated monoterpenoids (34.0%), fatty acids (30.3%), and oxygenated sesquiterpenoids (11.4%). The major components of the oil were 1-octene-3-ol (4.8%), linalool (1.9%), *endo*-fenchol (3.1%), camphor (4.4%), borneol (20.8%), eugenol (1.5%), lauric acid (2.3%), caryophyllene oxide (5.1%), myristic acid (3.3%), 6,10,14-trimethylpentadecane-2-one (3.0%), palmitic acid (16.0%), phytol (5.6%) and linoleic acid (7.0%) [69]. The oil showed some antibacterial activity against *Staphylococcus aureus* and *Bacillus subtilis* (MIC = 330 and 670 μg/mL, respectively). Moreover, it showed significant antioxidant activity in the DPPH- radical-scavenging assay (78% inhibition at 1.0 mg/mL).

#### 2.5.2. *Lavandula dentata* L.

The genus *Lavandula* consists of about 39 species [7] of which five are found growing naturally in Yemen [69]. In Yemeni folk medicine *Lavandula* species have been extensively used as a diuretic, an antiseptic, and for broncho-pulmonary infections [27]. *Lavandula dentata* is one of the species of *Lavandula* that are found growing in Yemen. It has been traditionally used in Yemeni folk medicine for the treatment of wounds, as a carminative, and to treat rheumatism [29]. *Lavandula dentata* from Yemen has been reported to have high concentrations of oxygenated monoterpenoids (51.8%), sesquiterpene hydrocarbons (13.9%), and oxygenated sesquiterpenoids (22.5%), with the major components being α-pinene (1.7%), camphene (1.8%), β-pinene (3.0%), fenchone (2.0%), linalool (3.7%), *endo*-fenchol (3.0%), *exo*-fenchol (2.4%), camphor (12.4%), *trans*-pinocarveol (7.5%), pinocarvone (3.1%), cryptone (3.0%), myrtenal (3.0%), myrtenol (3.9%), β-selinene (4.5%), caryophyllene oxide (3.1%), α-guiaol (6.1%), γ-eudesmol (2.4%), β-eudesmol (7.1%), and β-bisabolol (2.1%) [69]. The essential oil did not show any significant antimicrobial or antioxidant activity. 1,8-Cineole (41.3%) and sabinene (13.9%), sabinol (6.8%), and myrtenal (5.1%) were the major constituents of *Lavandula dentata* oil from Morocco [70]. Similarly, 1,8-cineole (33.5%), camphor (18.9%), and fenchone (8.4%), were major components found in *Lavandula dentata* oil from Tunisia [71]. The chemical composition of *Lavandula dentata* from Yemen differed qualitatively from samples growing in Morocco, Tunisia, Algeria and Saudi Arabia, but it most likely belongs to the camphor/*trans*-pinocarveol chemotype [69].

#### 2.5.3. *Lavandula pubescens* Decne.

*Lavandula pubescens* is known as “fahita” and has been traditionally used in Yemeni folk medicine as a carminative, insect repellent, and antiseptic [8]. *Lavandula pubescens* from Taiz, Yemen, is made up largely of carvacrol (20.6%), caryophyllene oxide (15.3%), β-bisabolene (12.0%), *p*-cymen-8-ol (11.8%), β-caryophyllene (10.7%), carvacrol methyl ether (7.2%), and terpinolene (6.0%) [15]. *Lavandula pubescens* essential oil has shown notable antibacterial activity against *Staphylococcus aureus* and *Salmonella enterica* (serovar Abony), and antifungal activity against *Aspergillus fumigatus* and *Candida albicans* [72]. The high concentration of carvacrol in this oil probably has some correlation to its use in folk medicine as an antiseptic, owing to the fact that the antimicrobial activity of carvacrol has been well established [73,74].

#### 2.5.4. *Leucas virgata* Balf. f.

There are around 100 species of *Leucas* distributed in Africa, Arabia, and Indomalaysia [7]. The Soqotra island of Yemen has 10 different species of *Leucas* of which *Leucas virgata* is endemic and known locally as “sa-lel hon”. It is an abundant aromatic shrub and grows up to 1 m in height [75]. The leaves and sprigs are used for tea and it has been traditionally used for the treatment of several stomach problems. The essential oil of *Leucas virgata* is rich in oxygenated monoterpenoids (50.8%), the major ones being camphor (20.5%), *exo-*fenchol (3.4%), fenchone (5.4%), and borneol (3.1%). It also has a high percentage of oxygenated sesquiterpenoids like β-eudesmol (6.1%) and caryophyllene oxide (5.1%). The essential oil exhibits very good antibacterial activity against *Staphylococcus aureus*, *Bacillus subtilis* and *Esherichia coli*. A methanolic extract of *Leucas virgata* has shown good activity against *Trypanosoma brucei* with an *IC*_50_ of 8.1 µg/mL [76]. *Leucas virgata* extracts have been reported to have moderate antimicrobial activity against various bacterial strains with MIC values ≤ to 250 µg/mL [62].

#### 2.5.5. *Mentha spicata* L.

*Mentha* is a comparatively small genus having 18–19 species [7]. It is a well-known genus for its medicinal properties and is found in the temperate regions of Eurasia, Australia, and South Africa, and is cultivated from tropical to temperate climates of America, Europe, China, Brazil, and India [77]. *Mentha spicata* (spearmint) is a perennial, rhizomatous and glabrous herb that has a strong aromatic odor. The spearmint odor of *Mentha spicata* comes from the high concentration of (*R*)-carvone present in it. There are various ethno-medicinal uses of *Mentha spicata* essential oil and the genus is one of the most researched for its components as well as for the biological activities. It has been extensively used for flatulence, acidity neutralization, gastro stimulation and digestive problems. It has also been used for cough and cold, as a diuretic and spasmolytic [78]. It is considered to have analgesic, antipyretic and anti-inflammatory effects as well [79]. The essential oil of *Mentha spicata* from the Sana’a region of Yemen was composed largely of carvone (63.0%), with lesser amounts of limonene (7.9%), 1,8-cineole (4.8%), terpinen-4-ol (2.9%), *cis*-dihydrocarvone (6.1%), and β-caryophyllene (2.4%) [16]. Carvone is the characteristic volatile component of *Mentha spicata* from which the plant species gets its distinct smell. There are various reports on the presence of carvone as the major component from *Mentha spicata* of various geographical origins.

The Yemeni oil sample was assayed for antimicrobial, antifungal and cytotoxicity against MCF-7 and MDA-MB-231 cells. The oil showed considerable activity against *Bacillus cereus*, *Esherichia coli*, and *Botrytis cinerea* with MIC of 312.5 µg/mL, 156 µg/mL and 78 µg/mL, respectively. However, the oil did not show any cytotoxic activity against either MCF-7 cells or MDA-MB-231 cells. Apart from these, *Mentha spicata* oil has been investigated for other biological activities like antioxidant activity, analgesic, anti-inflammatory and antipyretic effects. In a recent study, Liu and co-workers reported that *Mentha spicata* oil showed considerably strong cytotoxicity on HeLa cells with an *IC*_50_ of 2.08 µg/mL [80]. The oil was also reported to have high antibacterial effect against *Escherichia coli*, *Saccharomyces cerevisiae* and *Penicillium citrinum*. The major compounds in the essential oil were carvone (65.3%), limonene (18.2%), dihydrocarvone (3.0%) and camphene (2.3%). Agrawal and co-workers have examined the antimicrobial activities of both enantiomers of limonene and carvone [81]. These investigators found that both optical isomers of carvone showed activity against a wide spectrum of human pathogenic fungi and bacteria. Carvone has been found to inhibit the transformation of *Candida albicans* from a coccus to the filamentous form, so making them a potentially good therapeutic agent against infections caused by fungus. Other properties of carvone include its strong insect repellent activity and a promising sprouting inhibitory action on potatoes. (*S*)-carvone is a good potato sprout inhibitor, and is commercialized in the Netherlands under the name “Talent” [82].

#### 2.5.6. *Meriandra bengalensis* (Konig ex Roxb.) Benth.

*Meriandra bengalensis*, known as “dharo”, is an aromatic shrub that grows to a height of about 2 m and is highly branched. It has been used traditionally as an antiseptic, astringent, antirheumatic, and carminative [83]. *Meriandra bengalensis* from Yemen showed camphor (43.6%), 1,8-cineole (10.7%), borneol (3.4%), caryophyllene oxide (5.8%) and α-eudesmol (5.8%) as the major constituents [83]. Although the essential oil sample did not show significant antibacterial or antifungal activity, some of its major constituents do have biological activity. Camphor is considered to have good activity against human- and soil-born fungi. At a concentration of 500 µL/L, camphor has been shown to reduce the radial growth of *Sclerotinia sclerotiorum* and *Rhizoctonia solani* by 51.7% and 64.9%, respectively [84]. Camphor has been found to have good fungicidal activity against *Botrytis cinerea* where it showed complete inhibition of mycelia growth at 1.75 g/L [85].

#### 2.5.7. *Nepeta deflersiana* Schweinf. ex Hedge

*Nepeta deflersiana* is known locally as “mokerker alkotat”. It is an aromatic perennial herb traditionally used for wounds, as a carminative, for rheumatic disorders and for fever and colic [29]. Mothana examined *Nepeta deflersiana* essential oil from Sana’a and found the principle components to be palmitic acid (8.0%), caryophyllene oxide (6.4%), 2-methoxy-*p*-cresol (5.6%), camphor (4.7%), and eugenol (4.7%), but was devoid of nepetalactones [86]. This oil was weakly antibacterial against *Staphylococcus aureus* and *Bacillus subtilis* (MIC = 400 μg/mL), but was inactive against *Esherichia coli*, *Pseudomonas aeruginosa*, or *Candida albicans*. In contrast, another study has revealed *Nepeta* *deflersiana* essential oils from Taiz and from Sana’a to have very different compositions [16,87]. *Nepeta deflersiana* oil from Taiz was composed largely of germacrene D (40.5%), as well as nepetalactones, 4aα,7α,7aβ-nepetalactone (19.2%), 4aα,7β,7aα-nepetalactone (4.6%), and 4aα,7α,7aα-nepetalactone (3.0%), while an oil from Sana’a was dominated by 4aα,7α,7aα-nepetalactone (77.7%) with a smaller amount of germacrene D (6.0%). The oil from Taiz was somewhat antifungal against *Aspergillus niger* (MIC = 156 μg/mL) while the Sana’a sample was active against *Staphylococcus aureus* (MIC = 156 μg/mL) [16].

#### 2.5.8. *Ocimum basilicum* L.

The genus *Ocimum* is made up of around 65 species found throughout tropical and subtropical regions [7]. The genus is represented by seven species in Yemen, namely *Ocimum basilicum*, *Ocimum tenuiflorum*, *Ocimum suave*, *Ocimum spicatum*, *Ocimum gratissimum*, and *Ocimum forskolei*. *Ocimum basilicum* is used in Yemeni traditional medicine to treat various ailments, including abdominal cramps, gastroenteritis, dysentery, and diarrhea. In northern Oman and Saudi Arabia, juice of leaves or crushed leaves is used in the treatment of wounds, acne, and vitiligo. It is used also as a deodorant and is considered to be an aphrodisiac [27,88]. *Ocimum basilicum* from Yemen was dominated by linalool (74.5%) with lower concentrations of 1,8-cineole (7.4%) and estragole (7.2%) [16]. The Yemeni basil oil was screened for *in vitro* cytotoxic activity against MCF-7 (human mammary ductal carcinoma) cells, but was inactive; only 18.5% reduction of cell viability at a concentration of 100 μg/mL.

#### 2.5.9. *Origanum majorana* L.

*Origanum* is a perennial herbaceous genus that is native to North America, Europe, and temperate Asia [89]. The *Origanum* genus consists of over 44 species of which *Origanum majorana* (sweet marjoram), *Origanum vulgare* (oregano) and *Origanum maru* (Egyptian marjoram) are of medicinal importance in Yemen [90]. The genus is important medicinally due to its antimicrobial, antifungal, antioxidant, antibacterial, antithrombin, antimutagenic, angiogenic, antiparasitic, and antihyperglycemic activities. *Origanum majorana* has been used extensively throughout the world for its medicinal value and for its flavor properties. Dried leaves and flowering tips are used in formulation of vermouths and bitters. The essential oil is widely used for flavoring various food products such as sauces, condiments, and other products. It has great importance as a diuretic, anti-asthmatic, and an antipyretic drug in India. It has been used for the management of cancer as well [89]. The essential oil of *Origanum majorana*, known locally as “azab”, from the Al-Mahweet region of Yemen had sabinene (4.2%), *p*-cymene (9.8%), limonene (2.8%), γ-terpinene (7.7%), *cis*-sabinene hydrate (3.2%), *trans*-sabinene hydrate (6.8%), *cis*-*p*-menth-2-en-1-ol (2.3%), terpinen-4-ol (35.2%), α-terpineol (4.5%) and bornyl acetate (2.9%) as the major constituents [16].

#### 2.5.10. *Plectranthus barbatus* Andrews

*Plectranthus* is a large genus that belongs to the Lamiaceae and contains around 200 species of herbs and shrubs, most of which are aromatic plants tropical and sub-tropical areas of the Old World [7]. There are 12 species of *Plectranthus* found growing in Yemen, and this genus has been widely used for its medicinal properties. They have been used for treating skin, digestive, and respiratory complications [27,91]. *Plectranthus barbatus* is a perennial shrub that grows over the southern and subtropical regions of India, Pakistan, Sri Lanka, tropical east Africa, Asia (South of Arabian Peninsula), China, and Brazil [92]. The main constituents isolated from *Plectranthus barbatus* have been diterpenoids and essential oils. The essential oil composition varies according to the location and the date of harvest and contains mainly mono- and sesquiterpenes [92]. A wide range of biological activity has been reported for *Plectranthus* species. They are very important in ethno-medicinal uses to treat a range of ailments, particularly digestive, skin, infective, and respiratory problems. The plant has also been used widely for food, flavor and fodder.

*Plectranthus barbatus* is locally known as “baydat” in Yemen. The essential oil of *Plectranthus barbatus* from Taiz region of Yemen has been found to have α-terpinene (2.8%), *p*-cymene (9.3%), γ-terpinene (20.0%), thymol (48.7%), β-caryophyllene (6.4%) and β-selinene (2.1%) as the major constituents [16]. The chemical composition of Yemeni *Plectranthus barbatus* was very different from a *Plectranthus barbatus* sample from Portugal, which was devoid of thymol [93]. The Taiz oil sample was subjected to antimicrobial, antifungal, and cytotoxic assays against MCF-7 and MDA-MB-231 cancer cells. *Plectranthus barbatus* (Taiz) showed considerable activity against *Bacillus cereus* (MIC = 156 µg/mL) and *Esherichia coli* (MIC = 156 µg/mL). It also showed good activity against *Aspergillus niger* (MIC = 156 µg/mL) and *Botrytis cinerea* (MIC = 312.5 µg/mL). On the other hand, *Plectranthus barbatus* from the Ibb region of Yemen showed some activity against *Esherichia coli* (312.5 µg/mL). *Plectranthus barbatus* (Ibb) showed very good cytotoxic activity against MCF-7 cells (*IC*_50_ = 38.62 µg/mL) and had a 100% growth inhibition of MDA-MB-231 cells at concentration 100µg/mL [16]. The bioactivities of *Plectranthus barbatus* essential oils can be attributed to the high concentrations of thymol [74,94,95].

#### 2.5.11. *Plectranthus cylindraceus* Hochst. ex Benth.

*Plectranthus cylindraceus* is a strong aromatic plant found growing in different parts of United Arab Emirates, Saudi Arabia, Oman, Yemen, and East African countries. GC-MS analysis of the essential oil *Plectranthus cylindraceus* growing in Yemen showed high concentrations of thymol (68.5%), terpinolene (5.3%), β-selinene (4.7%), β-caryophyllene (4.0%), δ-cadinol (2.1%) and *ar*-curcumene (1.7%). The oil showed very good antibacterial and antifungal activity against *Staphylococcus aureus*, *Bacillus subtilis* and *Candida albicans* with MIC of 390, 180 and 180 µL/mL, respectively. It also showed considerable 2,2-diphenyl-1-picrylhydrazyl (DPPH) radical-scavenging activity as an antioxidant with *IC*_50_ of 34.5 µg/mL [83]. A similar study on *Plectranthus cylindraceus* oil from Oman has shown carvacrol (46.8%) and terpinolene (18.2%) to be the major constituents [96]. Note that thymol and carvacrol have similar retention indices on DB-1 or DB-5 columns and virtually identical mass spectra; these may be the same compound or overlapping mixtures of thymol and carvacrol in the *Plectranthus cylindraceus* samples. The oil also showed antimicrobial [96] and nematicidal activity [97]. The antibacterial and antifungal activity of *Plectranthus cylindraceus* is probably due to the presence of high concentration of thymol/carvacrol (see above).

#### 2.5.12. *Stachys yemenensis* Hedge

*Stachys* species are mostly distributed in the Mediterranean regions and Southwest Asia [7]. *Stachys* is represented by two species in Yemen, namely the endemic *S. yemenensis* [98] and *S. aegyptiaca* [99]. Many *Stachys* species have been used in folk medicine for the treatment of genital tumors, sclerosis of the spleen, inflammatory tumors and cancerous ulcers. A comparative study done on the *Stachys yemenensis* essential oil extracted by supercritical carbon dioxide extraction at 90 bar and by hydrodistillation [100] showed α-pinene (2.4%, 4.6%), α-phellandrene (13.9, 20.7%), *o*-cymene (5.3, 8.5%), β-phellandrene (11.7, 16.8%), bicyclogermacrene (4.3, 3.4%), elemol (12.0, 7.5%), spathulenol (6.7, 4.7%), γ-eudesmol (1.7, 3.2%), β-eudesmol (5.0, 5.1%), α-eudesmol (4.7, 6.4%), shyobunol (6.0, 1.5%) and squalene (4.9, 0%) as the major compounds. The exhausted matrix after the first supercritical extraction was further subjected to 250 bar supercritical CO_2_ extraction. The major constituents of the second run were elemol (5.9%), spathulenol (3.3%), γ-eudesmol (1.6%), β-eudesmol (5.1%), α-eudesmol (5.2%) and squalene (49.7%). The oil showed good antimicrobial activity against *E. coli* and *S. aureus*. Studies have shown that only the positive enantiomer of α-pinene is active. Time of kill curves have shown (+)-α-pinene to be highly toxic to *Candida albicans*, killing 100% of the inoculum within 60 min. It has also been reported to have synergistic activity against MRSA with commercial antibiotics like ciprofloxacin [101].

#### 2.5.13. *Teucrium yemense* Deflers

*Teucrium* has about 250 species [7] of which Yemeni flora are represented by three: *T. balfouri*, *T. sokotranum,* and *T. yemense* [102]. The genus plays an important role in Yemeni folk medicine, being used as insect repellant, antispasmodic, and for kidney disease, rheumatism and diabetes [45]. *T. yemense* is known locally as “khodas”, and the essential oil from Taiz, Yemen, has been reported to have high concentrations of sesquiterpene hydrocarbons (73.9%), the dominant ones being δ-cadinene (34.9%), β-caryophyllene (22.7%), α-humulene (6.1%), and α-selinene (5.4%) [102]. In another study, *T. yemense* from Taiz was reported to have α-pinene (6.6%), β-caryophyllene (19.1%), α-humulene (6.4%), δ-cadinene (6.5%), caryophyllene oxide (4.3%) and α-cadinol (9.5%) as the major constituents [87].

#### 2.5.14. *Thymus laevigatus* Vahl

*Thymus laevigatus* is an endemic species to Yemen and is the only species that represents this genus in Yemen [103]. The genus *Thymus* has about 250 species that are mostly perennial, aromatic and evergreen plants [7]. *Thymus laevigatus*, known locally as “zatar”, is mostly found in the higher mountains in North Yemen in Haggah (2500 m) and in Dhamar (2200 m). In Yemeni folk medicine, *Thymus laevigatus* dried leaves are used as powder in warm milk, sesame oil, or olive oil for the treatment of different stomach diseases, cough, tonsillitis, pharyngitis and renal colic [103]. The essential oils from many species of *Thymus* have been reported to have very good antibacterial and antifungal activities, which are attributed to the high concentrations of thymol and carvacrol is those oils [104,105]. An essential oil study on *Thymus laevigatus* from Yemen has shown a very high concentration of carvacrol (84.3%), along with *p*-cymene (4.1%), γ-terpinene (4.0%) and *trans*-anethole (3.6%) as the major constituents [103]. *Thymus laevigatus* oil was found to have a wide range of antimicrobial activity and a potent fungicidal effect against *Candida albicans* with a MIC of 0.0313% (*v*/*v*) [104].

## 3. Conclusions

Yemen is a land of diverse landscapes including mountains, desert, gorge-like wadis, and coastal escarpments. The variety of habitats of mainland Yemen, coupled with the endemism of the Soqotra archipelago, give Yemeni flora a great deal of diversity. There are currently around 2930 species of higher plants in Yemen [106,107] of which 699 species are endemic [108]. Despite the introduction of Western medicinal system during the middle of the twentieth century, traditional herbal medicine continues to play an important role in many parts of Yemen. Many of these medicinal plants and their essential oils have shown notable biological activities. In addition to research activities on these traditional medicinal plants, it is hoped that future studies will provide new insights into pharmacological activities of understudied Yemeni flora. Unfortunately, increasing environmental degradation due to human activity, invasive plant species, and climate change threaten the native flora of Yemen. It is hoped that steps be undertaken to safeguard the fragile ecology of Yemen, protect the native flora and fauna, as well as preserve the traditional knowledge of the people.

## References

[1] Bakkali F., Averbeck S., Averbeck D., Idaomar M. (2008). Biological effects of essential oils—A review. Food Chem. Toxicol..

[2] Christaki E., Bonos E., Giannenas I., Paneri P.F. (2012). Aromatic plants as a source of bioactive compounds. Agriculture.

[3] Dresch P. (2000). A History of Modern Yemen.

[4] Hadden R.L. (2012). The Geology of Yemen: An Annotated Bibliography of Yemen’s Geology, Geography and Earth Science.

[5] Turek C., Stintzing F.C. (2013). Stability of essential oils: A review. Compr. Rev. Food Sci. Food Saf..

[6] Shaaban H.A.E., El-Ghorab A.H., Shibamoto T. (2012). Bioactivity of essential oils and their volatile aroma components: Review. J. Essent. Oil Res..

[7] Mabberley D.J. (2008). Mabberley’s Plant-Book.

[8] Dubai A., Alkhulaidi A. (1997). Medicinal and Aromatic Plants in Yemen.

[9] Gundidza M. (1993). Antimicrobial activity of essential oil from *Schinus molle* Linn. Cent. Afr. J. Med..

[10] Díaz C., Quesada S., Brenes O., Aguilar G., Cicció J.F. (2008). Chemical composition of *Schinus molle* essential oil and its cytotoxic activity on tumour cell lines. Nat. Prod. Res..

[11] Abdel-Sattar E., Zaitoun A.A., Farag M.A., El Gayed S.H., Harraz F.M.H. (2010). Chemical composition, insecticidal and insect repellent activity of *Schinus molle* L. leaf and fruit essential oils against *Trogoderma granarium* and *Tribolium castaneum*. Nat. Prod. Res..

[12] Ali N.A.A., Marongiu B., Piras A., Porcedda S., Falconieri D., Al-Othman A.M.R. (2011). Comparative analysis of the oil and supercritical CO_2_ extract of *Schinus molle* L. growing in Yemen. Nat. Prod. Res..

[13] Schmidt J.M., Noletto J.A., Vogler B., Setzer W.N. (2007). Abaco bush medicine: Chemical composition of the essential oils of four aromatic medicinal plants from Abaco Island, Bahamas. J. Herbs Spices Med. Plants.

[14] Mossa J.S. (1985). Phytochemical and biological studies on *Artemisia abyssinica* and anti diabetic herb used in Arabian folk medicine. Fitoterapia.

[15] Abad J.M., Bedoya L.M., Apaza L., Bermejo P. (2012). The *Artemisia* L. genus: A review of bioactive essential oils. Molecules.

[16] Chhetri B.K. (2015). A Gas Chromatographic/Mass Spectral Analysis of Aromatic Medicinal Plants from Yemen. M.S. Thesis.

[17] Chhetri B.K., Al-Sokari S.S., Setzer W.N., Ali N.A.A. (2015). Essential oil composition of *Artemisia abyssinica* from three habitats in Yemen. Am. J. Essent. Oils Nat. Prod..

[18] Tariku Y., Hymete A., Hailu A., Rohloff J. (2010). Essential-oil composition, antileishmanial, and toxicity study of *Artemisia abyssinica* and *Satureja punctata* ssp. *punctata* from Ethiopia. Chem. Biodivers..

[19] Militello M., Settanni L., Aleo A., Mammina C., Moschetti G., Giammanco G.M., Amparo Blàzquez M., Carrubba A. (2011). Chemical composition and antibacterial potential of *Artemisia arborescens* L. essential oil. Curr. Microbiol..

[20] Saddi M., Sanna A., Cottiglia F., Chisu L., Casu L., Bonsignore L., de Logu A. (2007). Antiherpesvirus activity of *Artemisia orborescens* essential oil and inhibition of lateral diffusion in Vero cells. Ann. Clin. Microbiol. Antimicrob..

[21] Ali N.A.A., Wurster M., Denkert A., Al-Sokari S.S., Lindequist U., Wessjohann L. (2014). Cytotoxicity and antiphytofungal activity of the essential oils from two *Artemisia* species. World J. Pharm. Res..

[22] Magnelli L., Caldini R., Schiavone N., Suzuki H., Chevanne M. (2010). Differentiating and apoptotic dose-dependent effects in (-)-α-bisabolol-treated human endothelial cells. J. Nat. Prod..

[23] Siegel I., Liu T.L., Yaghoubzadeh E., Keskey T.S., Gleicher N. (1987). Cytotoxic effects of free fatty acids on ascites tumor cells. J. Nat. Cancer Inst..

[24] Abderrahim A., Belhamel K., Cahlchat J.C., Figuérédo G. (2010). Chemical composition of the essential oil from *Artemisia arborescens* L. growing wild in Algeria. Rec. Nat. Prod..

[25] Thabit R.A.S., Cheng X., Al-Hajj N., Rahman M.R.T., Lei G. (2014). Antioxidant and *Conyza bonariensis*: A review. Eur. Acad. Res..

[26] Cheng X.R., Thabit R.A., Wang W., Shi H.W., Shi Y.H., Le G.W. (2013). Analysis and comparison of the essential oil in *Conyza bonariensis* grown in Yemen and China. Prog. Mod. Biomed..

[27] Ghazanfar S.A. (1994). Handbook of Arabian Medicinal Plants.

[28] Flann C., Roskov Y., Kunze T., Paglinawan L., Orrell T., Nicolson D., Culham A., Bailly N., Kirk P., Bourgoin T., Baillargeon G. GCC: Global Compositae Checklist (version 5 (Beta), June 2014). Species 2000 & ITIS Catalogue of Life, 15th February 2015.

[29] Mothana R.A.A., Gruenert R., Bednarski P.J., Lindequist U. (2009). Evaluation of the *in vitro* anticancer, antimicrobial and antioxidant activities of some Yemeni plants used in folk medicine. Pharmazie.

[30] Al-Hajj N.Q.M., Ma C., Thabit R., Al-alfarga A., Gasmalla M.A.A., Musa A., Aboshora W., Wang H. (2014). Chemical composition of essential oil and mineral contents of *Pulicaria inuloides*. J. Acad. Indust. Res..

[31] Al-Hajj N.Q.M., Wang H.X., Ma C., Lou Z., Bashari M., Thabit R. (2014). Antimicrobial and antioxidant activities of the essential oils of some aromatic medicinal plants (*Pulicaria inuloides*–Asteraceae and *Ocimum forskolei*–Lamiaceae. Trop. J. Pharm. Res..

[32] Fawzy G.A., Al Ati H.Y., El Gamal A.A. (2013). Chemical composition and biological evaluation of essential oils of *Pulicaria jaubertii*. Pharmacogn. Mag..

[33] Algabr M.N., Ameddah S., Menad A., Mekkiou R., Chalchat J.C., Benayache S., Benayache F. (2012). Essential oil composition of *Pulicaria jaubertii* from Yemen. Int. J. Med. Aromat. Plants.

[34] Ali N.A.A., Crouch R.A., Al-Fatimi M.A., Arnold N., Teichert A., Setzer W.N., Wessjohann L. (2012). Chemical composition, antimicrobial, antiradical and anticholinesterase activity of the essential oil of *Pulicaria stephanocarpa* from Soqotra. Nat. Prod. Commun..

[35] Ali N.A.A., Al-Haj M.A., Wurster M., Lindequist U. (2009). Chemical composition and antifungal activity of essential oil of Soqotran *Pulicaria stephanocarpa* Balf. f.. Univ. Aden J. Nat. Appl. Sci..

[36] Hammiche V., Maiza K. (2006). Traditional medicine in Central Sahara: Pharmacopoeia of Tassili N’ajjer. J. Ethnopharmacol..

[37] Hegazy M.E.F., Matsuda H., Nakamura S., Yabe M., Matsumoto T., Yoshikawa M. (2012). Sesquiterpenes from an Egyptian herbal medicine, *Pulicaria undulata*, with inhibitory effects on nitric oxide production in RAW264.7 macrophage cells. Chem. Pharm. Bull..

[38] El-Kamali H.S., Ahmed A.H., Mohammed A.S., Yahia A.A.M., El-Tayeb I.H., Ali A.A. (1998). Antibacterial properties of essential oils from *Nigella sativa* seeds, *Cymbopogon citratus* leaves and *Pulicaria undulata* aerial parts. Fitoterapia.

[39] Ali N.A.A., Makboul M.A., Assaf M.H., Anton R. (1987). Essential oil of *Pulicaria undulata* L. growing in Egypt and its effect on animal behaviour. Bull. Pharm. Sci..

[40] Elegami A.A.B., Ishag K.E., Mahmoud E.N., Abu Alfutuh I.M., Karim E.I.A. (1994). Insecticidal activity of *Pulicaria undulata* oil. Fitoterapia.

[41] Ali N.A.A., Sharopov F.S., Alhaj M., Hill G.M., Porzel A., Arnold N., Setzer W.N., Schmidt J., Wessjohann L. (2012). Chemical composition and biological activity of essential oil from *Pulicaria undulata* from Yemen. Nat. Prod. Commun..

[42] El-Kamali H.H., Yousif M.O., Ahmed O.I., Sabir S.S. (2009). Phytochemical analysis of the essential oil from aerial parts of *Pulicaria undulata* (L.) Kostel from Sudan. Ethnobot. Leaf..

[43] Soule J.A., Janick J., Simon J.E. (1993). *Tagetes minuta*: A potential new herb from South America. New Crops.

[44] Duke J.A., Bogenschutz-Godwin M.J., Ottesen A.R. (2009). Duke’s Handbook of Medicinal Plants of Latin America.

[45] Al-Musayeib N.M., Mothana R.A., Matheeussen A., Cos P., Maes L. (2012). *In vitro* antiplasmodial, antileishmanial and antitrypanosomal activities of selected medicinal plants used in the traditional Arabian Peninsular region. BMC Complement. Altern. Med..

[46] Bii C.C., Siboe G.M., Mibey R.K. (2000). Plant essential oils with promising antifungal activity. East Afr. Med. J..

[47] Senatore F., Napolitano F., Mohamed M.A., Harris H.P.J.C., Mnkeni P.N.S., Henderson J. (2004). Antibacterial activity of *Tagetes minuta* L. (Asteraceae) essential oil with different chemical composition. Flavour Fragr. J..

[48] Chamorro E.R., Sequeira A.F., Velasco G.A., Zalazar M.F., Ballerini J. (2011). Evaluation of *Tagetes minuta* L. essential oils to control *Varroa destructor* (Acari: Varroidae). J. Argent. Chem. Soc..

[49] López S.B., López M.L., Aragón L.M., Tereschuck M.L., Slanis A.C., Feresin G.E., Zygadlo J.A., Tapia A.A. (2011). Composition and anti-insect activity of essential oils from *Tagetes* L. species (Asteraceae, Helenieae) on *Ceratitis capitata* Wiedemann and *Triatoma infestans* Klug. J. Agric. Food Chem..

[50] Garcia M.V., Matias J., Barros J.C., de Lima D.P., Lopes Rda.S., Andreotti R. (2012). Chemical identification of *Tagetes minuta* Linneus (Asteraceae) essential oil and its acaricidal effect on ticks. Rev. Bras. Parasitol. Vet..

[51] Ali N.A.A., Sharopov F.S., Al-kaf A.G., Hill G.M., Arnold N., Al-Sokari S.S., Setzer W.N., Wessjohann L. (2014). Composition of essential oil from *Tagetes minuta* and its cytotoxic, antioxidant and antimicrobial activities. Nat. Prod. Commun..

[52] Al-Mamary M., Abdelwahab S.I., Al-Ghalibi S., Al-Ghasani E. (2011). The antioxidant and tyrosinase inhibitory activities of some essential oils obtained from aromatic plants grown and used in Yemen. Sci. Res. Essays.

[53] Ali N.A.A., Al-Fatimi M.A., Crouch R.A., Denkert A., Setzer W.N. (2013). Antimicrobial, antioxidant, and cytotoxic activities of the essential oil of *Tarchonanthus camphoratus*. Nat. Prod. Commun..

[54] Miller G.A., Morris M. (2004). Ethnoflora of the Soqotra Archipelago.

[55] De Sanctis M., Adeeb A., Farcomeni A., Patriarca C., Saed A., Attorre F. (2012). Classification and distribution patterns of plant communities on Socotra Island, Yemen. Appl. Veget. Sci..

[56] Ali N.A.A., Wurster M., Arnold N., Teichert A., Schmidt J., Lindequist U., Wessjohann L. (2008). Chemical composition and biological activities of essential oils from the oleogum resins of three endemic Soqotraen *Boswellia* species. Rec. Nat. Prod..

[57] Mothana R.A.A., Lindequist U. (2005). Antimicrobial activity of some medicinal plants of the island of Soqotra. J. Ethnopharmacol..

[58] Ali N.A.A., Wurster M., Lindequist U. (2009). Chemical composition of essential oil from the oleogum resin of *Commiphora habessinica* (Berg.) Engl. from Yemen. J. Essent. Oil Bear. Plants.

[59] Banfield L.M., Van Damme K., Miller A.G., Bramwell D., Caujapé-Castells J. (2011). Evolution and biogeography of the flora of the Socotra archipelago (Yemen). The Biology of Island Floras.

[60] Ali N.A.A., Wurster M., Arnold N., Lindequist U., Wessjohann L. (2008). Essential oil composition from oleogum resin of Soqotraen *Commiphora kua*. Rec. Nat. Prod..

[61] Al-Fatimi M., Wurster M., Schröder G., Lindequist U. (2007). Antioxidant, antimicrobial and cytotoxic activities of selected medicinal plants from Yemen. J. Ethnopharmacol..

[62] Mothana R.A., Lindequist U., Gruenert R., Bednarski P.J. (2009). Studies of the *in vitro* anticancer, antimicrobial and antioxidant potentials of selected Yemeni medicinal plants from the island Soqotra. BMC Complement. Altern. Med..

[63] Mothana R.A., Al-Rehaily A.J., Schultze W. (2010). Chemical analysis and biological activity of the essential oils of two endemic Soqotri *Commiphora* species. Molecules.

[64] Zahran M.A., Khedr A., Dahmash A., El-Ameir Y.A. (2014). Qat forms in Yemen: Ecology, dangerous impacts and future promise. Egypt J. Basic Appl. Sci..

[65] Kalix P. (1992). Cathinone, a natural amphetamine. Pharmacol. Toxicol..

[66] Baxter R.L., Crombie L., Simmonds D.J., Whiting D.A., Braenden O.J., Szendrei K. (1979). Alkaloids of *Catha edulis* (khat). Part 1. Isolation and characterization of eleven new alkaloids with sesquiterpene cores (cathedulins); identification of the quinone-methide root pigments. J. Chem. Soc., Perkin Trans. 1.

[67] Algabr M.N., Al-Wadhaf H.A., Ameddah S., Menad A., Mekkiou R., Chalchat J.C., Benayache S., Benayache F. (2014). Analysis of the essential oil of *Catha edulis* leaves from Yemen. Int. J. Appl. Res. Nat. Prod..

[68] Adorjan B., Buchbauer G. (2010). Biological properties of essential oils: An updated review. Flavour Fragr. J..

[69] Mothana R.A., Alsaid M.S., Hasoon S.S., Al-Mosaiyb N.M., Al-Rehaily A.J., Al-Yahya M.A. (2012). Antimicrobial and antioxidant activities and gas chromatography mass spectrometry (GC/MS) analysis of the essential oils of *Ajuga brancteosa* Wall. ex Benth. and *Lavandula dentata* L. growing wild in Yemen. J. Med. Plants Res..

[70] Imelouane B., Elbachiri A., Ankit M., Benzeid H., Khedid K. (2009). Physico-chemical compositions and antimicrobial activity of essential oil of eastern Moroccan *Lavandula dentata*. Int. J. Agric. Biol..

[71] Touati R., Chograni H., Hassen I., Boussaïd M., Toumi L., Brahim N.B. (2011). Chemical composition of the leaf and flower essential oils of Tunisian *Lavandula dentata* L. (Lamiaceae). Chem. Biodivers..

[72] Alkhyat S.H., Maqtari M.A.A., Alhamzy E.H., Saeed M.A., Ali N.A.A. (2014). Antimicrobial activity of *Lavandula pubescens* essential oil from two places In Yemen. J. Adv. Biol..

[73] Cantore P.L., Shanmugaiah V., Iacobellis N.S. (2009). Antibacterial activity of essential oil components and their potential use in seed disinfection. J. Agric. Food Chem..

[74] Marei G.I.K., Rasoul M.A.A., Abdelgaleil S.A.M. (2012). Comparative antifungal activities and biochemical effects of monoterpenes on plant pathogenic fungi. Pestic. Biochem. Physiol..

[75] Mothana R.A., Al-Said M., Al-Yahya M.A., Al-Rehaily A.J., Khaled J.M. (2013). GC and GC/MS analysis of essential oil composition of the endemic Soqotraen *Leucas virgata* Balf.f. and its antimicrobial and antioxidant activities. Int. J. Mol. Sci..

[76] Mothana R.A., Al-Musayeib N.M., Al-Ajmi M.F., Cos P., Maes L. (2014). Evaluation of the *In vitro* antiplasmodial, antileishmanial, and antitrypanosomal activity of medicinal plants used in Saudi and Yemeni traditional medicine. Evid.-Based Complement. Altern. Med..

[77] Chauhan R.S., Kaul M.K., Shahi A.K., Kumar A., Ram G., Tawa A. (2009). Chemical composition of essential oils in *Mentha spicata* L. accession [IIIM(J)26] from North-West Himalayan region, India. Ind. Crops Prod..

[78] Karousou R., Balta M., Hanlidou E., Kokkini S. (2007). “Mints”, smells and traditional uses in Thessaloniki (Greece) and other Mediterranean countries. J. Ethnopharmacol..

[79] Yousuf P.M.H., Noba N.Y., Shohel M., Bhattacherjee R., Das B.K. (2013). Analgesic, anti-Inflammatory and antipyretic effect of *Mentha spicata* (spearmint). Br. J. Pharm. Res..

[80] Liu K.H., Zhu Q., Zhang J.J., Xu J.F., Wang X.C. (2012). Chemical composition and biological activities of the essential oil of *Mentha spicata* Lamiaceae. Adv. Mater. Res..

[81] Aggarwal K.K., Khanuja S.P.S., Ahmad A., Kumar T.R.S., Gupta V.K., Kumar S. (2002). Antimicrobial activity profiles of the two enantiomers of limonene and carvone isolated from the oils of *Mentha spicata* and *Anethum sowa*. Flavour Fragr. J..

[82] De Carvalho C., da Fonseca M.M.R. (2006). Carvone: Why and how should one bother to produce this terpene. Food Chem..

[83] Ali N.A.A., Wurster M., Denkert A., Arnold N., Fadail I., Al-Didamony G., Lindequist U., Wessjohann L., Setzer W.N. (2012). Chemical composition, antimicrobial, antioxidant and cytotoxic activity of essential oils of *Plectranthus cylindraceus* and *Meriandra benghalensis* from Yemen. Nat. Prod. Commun..

[84] Pitarokili D., Tzakou O., Loukis A., Harvala C. (2003). Volatile metabolites from *Salvia fruticosa* as antifungal agents in soilborne pathogens. J. Agric. Food Chem..

[85] Moretti M.D.L., Peana A.T. (1996). Activity of the oil of *Salvia officinalis* L. against *Botrytis cinerea*. J. Essent. Oil Res..

[86] Mothana R.A. (2012). Chemical composition, antimicrobial and antioxidant activities of the essential oil of *Nepeta deflersiana* growing in Yemen. Rec. Nat. Prod..

[87] Maqtari M.A.A., Alhamzy E.H., Saeed M A., Ali N.A.A., Setzer W.N. (2014). Chemical composition and antioxidant activity of the essential oils from different aromatic plants grown in Yemen. J. Glob. Biosci..

[88] Schopen A. (1983). Traditionelle Heilmittel in Jemen.

[89] Chishti S., Kaloo Z.A., Sultan P. (2013). Medicinal importance of genus *Origanum*: A review. J. Pharmacogn. Phytother..

[90] Lev E., Hehmeyer I., Schönig H. (2012). Eastern Mediterranean pharmacology and India trade as a background for Yemeni medieval medicinal plants. Herbal Medicine in Yemen: Traditional Knowledge and Practice, and Their Value for Today’s World.

[91] Lukhoba C.W., Simmonds M.S.J., Paton A.J. (2006). *Plectranthus*: A review of ethnobotanical uses. J. Ethnopharmacol..

[92] Alasbahi R.H., Melzig M.F. (2010). *Plectranthus barbatus*: A review of phytochemistry, ethnobotanical uses and pharmacology—Part 1. Planta Med..

[93] Mota L., Figueiredo A.C., Pedro L.G., Barroso J.G., Miguel M.G., Falerio M.L., Ascensão L. (2014). Volatile-oils composition, and bioactivity of the essential oils of *Plecranthus barbatus*, *P. neochilus* and *P. ornatus* grown in Portugal. Chem. Biodivers..

[94] Gallucci M.N., Oliva M., Casero C., Dambolena J., Luna A., Zygadlo J., Demo M. (2009). Antimicrobial combined action of terpenes against the food-borne microorganisms *Escherichia coli*, *Staphylococcus aureus* and *Bacillus cereus*. Flavour Fragr. J..

[95] Sobral M.V., Xavier A.L., Lima T.C., de Sousa D.P. (2014). Antitumor activity of monoterpenes found in essential oils. Sci. World J..

[96] Marwah R.G., Fatope M.O., Deadman M.L., Ochei J.E., Al-Saidi S.H. (2007). Antimicrobial activity and the major components of the essential oil of *Plectranthus cylindraceus*. J. Appl. Microbiol..

[97] Onifade A.K., Fatope M.O., Deadman M.L., Al Kindy S.M. (2008). Nematicidal activity of *Haplophyllum tuberculatum* and *Plectranthus cylindraceus* oils against *Meloidogyne javanica*. Biochem. Syst. Ecol..

[98] Wood J.R.I. (1997). A Handbook of the Yemen Flora.

[99] Trease G.E., Evans W.C. (1989). Trease and Evans’ Pharmacognosy.

[100] Ali N.A.A., Marongiu B., Piras A., Porcedda S., Falconieri D., Molicotti P., Zanetti S. (2010). Essential oil composition of leaves of *Stachys yemenensis* obtained by supercritical CO_2_. Nat. Prod. Res..

[101] Da Silva A.C.R., Lopes P.M., de Azevedo M.M.B., Costa D.C.M., Alviano C.S., Alviano D.S. (2012). Biological activities of α-pinene and β-pinene enantiomers. Molecules.

[102] Ali N.A.A., Wurster M., Arnold N., Lindequist U., Wessjohan L. (2008). Chemical composition of the essential oil of *Teucrium yemense* Deflers. Rec. Nat. Prod..

[103] Al-Fatimi M., Wurster M., Schröder G., Lindequist U. (2010). *In vitro* antimicrobial, cytotoxic and radical scavenging activities and chemical constituents of the endemic *Thymus laevigatus* (Vahl). Rec. Nat. Prod..

[104] Othman A.M., Awadh N.A., Al-Fadhli E., Salama M. (2014). Topical herbal antimicrobial formulation containing *Thymus laevigatus* essential oil. World J. Pharm. Res..

[105] Loziene K. (2009). Selection of fecund and chemically valuable clones of thyme (*Thymus*) species growing wild in Lithuania. Ind. Crops Prod..

[106] Kilian N., Hein P., Hubaishan M.A. (2002). New and noteworthy records for the flora of Yemen, chiefly of Hadhramout and Al-Mahra. Willdenowia.

[107] Kilian N., Hein P., Hubaishan M.A. (2004). Further notes on the flora of the southern coastal mountains of Yemen. Willdenowia.

[108] Hall M., Miller A.G. (2011). Strategic requirements for plant conservation in the Arabian Peninsula. Zool. Middle East.

